# G Protein Beta 5 Is Targeted to D2-Dopamine Receptor-Containing Biochemical Compartments and Blocks Dopamine-Dependent Receptor Internalization

**DOI:** 10.1371/journal.pone.0105791

**Published:** 2014-08-27

**Authors:** J. Christopher Octeau, Joseph M. Schrader, Ikuo Masuho, Meenakshi Sharma, Christopher Aiudi, Ching-Kang Chen, Abraham Kovoor, Jeremy Celver

**Affiliations:** 1 Department of Biomedical and Pharmaceutical Sciences, University of Rhode Island, Kingston, Rhode Island, United States of America; 2 Department of Neuroscience, The Scripps Research Institute, Jupiter, Florida, United States of America; 3 Cullen Eye Institute, Baylor College of Medicine, Houston, Texas, United States of America; University of Oldenburg, Germany

## Abstract

G beta 5 (Gbeta5, Gβ5) is a unique G protein β subunit that is thought to be expressed as an obligate heterodimer with R7 regulator of G protein signaling (RGS) proteins instead of with G gamma (Gγ) subunits. We found that D2-dopamine receptor (D2R) coexpression enhances the expression of Gβ5, but not that of the G beta 1 (Gβ1) subunit, in HEK293 cells, and that the enhancement of expression occurs through a stabilization of Gβ5 protein. We had previously demonstrated that the vast majority of D2R either expressed endogenously in the brain or exogenously in cell lines segregates into detergent-resistant biochemical fractions. We report that when expressed alone in HEK293 cells, Gβ5 is highly soluble, but is retargeted to the detergent-resistant fraction after D2R coexpression. Furthermore, an in-cell biotin transfer proximity assay indicated that D2R and Gβ5 segregating into the detergent-resistant fraction specifically interacted in intact living cell membranes. Dopamine-induced D2R internalization was blocked by coexpression of Gβ5, but not Gβ1. However, the same Gβ5 coexpression levels had no effect on agonist-induced internalization of the mu opioid receptor (MOR), cell surface D2R levels, dopamine-mediated recruitment of β-arrestin to D2R, the amplitude of D2R-G protein coupling, or the deactivation kinetics of D2R-activated G protein signals. The latter data suggest that the interactions between D2R and Gβ5 are not mediated by endogenously expressed R7 RGS proteins.

## Introduction

The D2-dopamine receptor (D2R), is a G protein coupled receptor (GPCR) that is a major target of drugs used to alleviate symptoms of schizophrenia, Parkinson’s disease and depression [1], [2]. Many of the cellular actions of GPCRs are mediated via the activation of intracellular heterotrimeric G proteins, which consist of a Gα subunit and a protein dimer consisting of Gβ and γ subunits. When an activated GPCR encounters a trimeric G protein, it catalyzes the exchange of guanosine-5′-triphosphate (GTP) for guanosine diphosphate (GDP) at Gα, leading to the dissociation Gα subunit from a G protein beta-gamma dimer (Gβγ). The activated GTP-bound Gα subunit and the free Gβγ dimer regulate the activity of diverse cellular effector molecules. Signal termination is mediated by the intrinsic guanosine-5′-triphosphatase (GTPase) activity of the Gα, which hydrolyzes the bound GTP to GDP, allowing it to re-associate with the Gβγ dimer [3], [4].

Five different G protein Gβ subunits have been identified thus far, of which the first four share 80–90% homology. The fifth, Gβ5, is an atypical member, and shares only about 50% sequence homology with the first four members [5]. Two alternatively spliced isoforms of Gβ5 have been described. The “short” isoform (Gβ5) is broadly expressed in neural, neuroendocrine and other excitable tissues such as heart muscle [5], [6], while the long isoform (Gβ5L) has only been found expressed in retinal photoreceptors. Severe phenotypes associated with the Gβ5 knockout mice, indicate Gβ5 likely has many important and diverse cellular functions. For example, Gβ5 knockout mice have impaired brain development and exhibit multiple neurological abnormalities [7]–[9]. In addition, these mice have altered metabolism and abnormal weight regulation, presumably via actions in the central nervous system [7].

The GTPase activity of Gα G proteins is enhanced by RGS (regulator of G protein signaling) proteins and thus RGS proteins accelerate the rate of GPCR signal termination. All RGS proteins have a conserved core “RGS domain” which is necessary and sufficient for their GTPase accelerating protein (GAP) function [10]. Many RGS proteins also possess additional C- and N-terminal domains [11], [12] that mediate diverse functions.

For example, R7 RGS family proteins contain a Gγ-like (GGL) domain that has been shown to specifically bind Gβ5 subunits and enhance GAP function [5], [13]–[16]. In fact, it is thought that *in vivo*, Gβ5 does not form G protein Gβγ dimers, and that complex formation between Gβ5 and the Gγ-like domain-containing R7 RGS proteins is necessary for stabilizing both Gβ5 and R7 RGS proteins [5], [13]–[16]. The genetic ablation of Gβ5 resulted in the loss of all R7 RGS proteins [14], and conversely, Gβ5 protein was not detected in the retina of a triple knockout mouse line lacking the R7 RGS proteins, RGS6, RGS7, and RGS11 [17]. Furthermore, the Gβ5 long isoform (Gβ5l) that forms a complex with the R7 RGS protein, RGS9-1, was absent from the photoreceptors of RGS9 knockout mice [18].

However, it has not been proven that Gβ5 exists solely as a heterodimer with R7 RGS proteins in all tissues where Gβ5 may be expressed. Alternative proteins, not abundantly expressed in retinal cells, could contribute to stabilizing Gβ5 expression in other regions.

Complexes of Gβ5 and R7 RGS proteins can target to D2R and other GPCRs but these interactions are thought to occur through protein domains, such as the DEP domain, that are present within R7 RGS proteins [19]–[23].

We previously showed that significant proportion of cellular D2R segregates into a biochemical fraction that is resistant to solubilization in non-ionic detergents [24]. Subsequently, we utilized a novel in-cell biotin-transfer assay to demonstrate that this detergent-resistant D2R fraction originated from plasma membrane micro-compartments that existed in the living cell to restrict the accessibility of the resident D2R to other cellular proteins [25]. Conversely, the D2R that segregated into the detergent-soluble fraction originated from plasma membrane regions that allowed the D2R molecules to interact in a relatively unrestricted manner with other cellular proteins.

Here we report that the coexpression of D2R causes Gβ5 to target to the detergent-resistant cellular fractions and stabilizes Gβ5 to enhance Gβ5 expression. Moreover, the D2R-Gβ5 interaction likely occurs independently of R7 RGS proteins suggesting that Gβ5 may have additional cellular functions in addition to its established role as a component of the R7-RGS/Gβ5 complex.

## Results

### Coexpression of D2R in HEK293 cells enhances the detergent-resistance of Gβ5 even in the absence of exogenous coexpression of R7 RGS proteins

We had previously shown that the vast majority of D2-dopamine receptors (D2R) expressed endogenously in the brain, or exogenously in the plasma membrane of cell lines, segregates into cellular fractions that are resistant to solubilization in non-ionic detergents [24] and we showed that this detergent-resistant D2R fraction is functional and responds to dopamine [25]. Furthermore, coexpression of D2R produces translocation of putative D2R interacting proteins, such as the RGS9-2/Gβ5 complex and G proteins, from detergent-soluble to detergent-resistant membrane fractions when the latter proteins are expressed in cell lines [24], [25]. In fact, interactions with D2R, which is expressed at relatively high concentrations in the striatum compared to the cortex [1], [2], could explain why we and others have found that the endogenous striatal RGS9-2/Gβ5 complex is resistant to detergent-extraction, but the same complex when expressed in cell lines is highly soluble [24], [31].

To provide further support for the idea that targeting to D2R can contribute to enhanced detergent resistance of D2R-interacting proteins the striatum, we compared the detergent-solubility of Gβ5 endogenously expressed in mouse striatum and the cortex. We found that the percent of striatal Gβ5 that was extracted into cold solutions (4°C) of the non-ionic detergent Triton X-100 was almost halved (from ∼40% to 20%), relative to Gβ5 extracted from the cortex [24].

**Figure 1:**
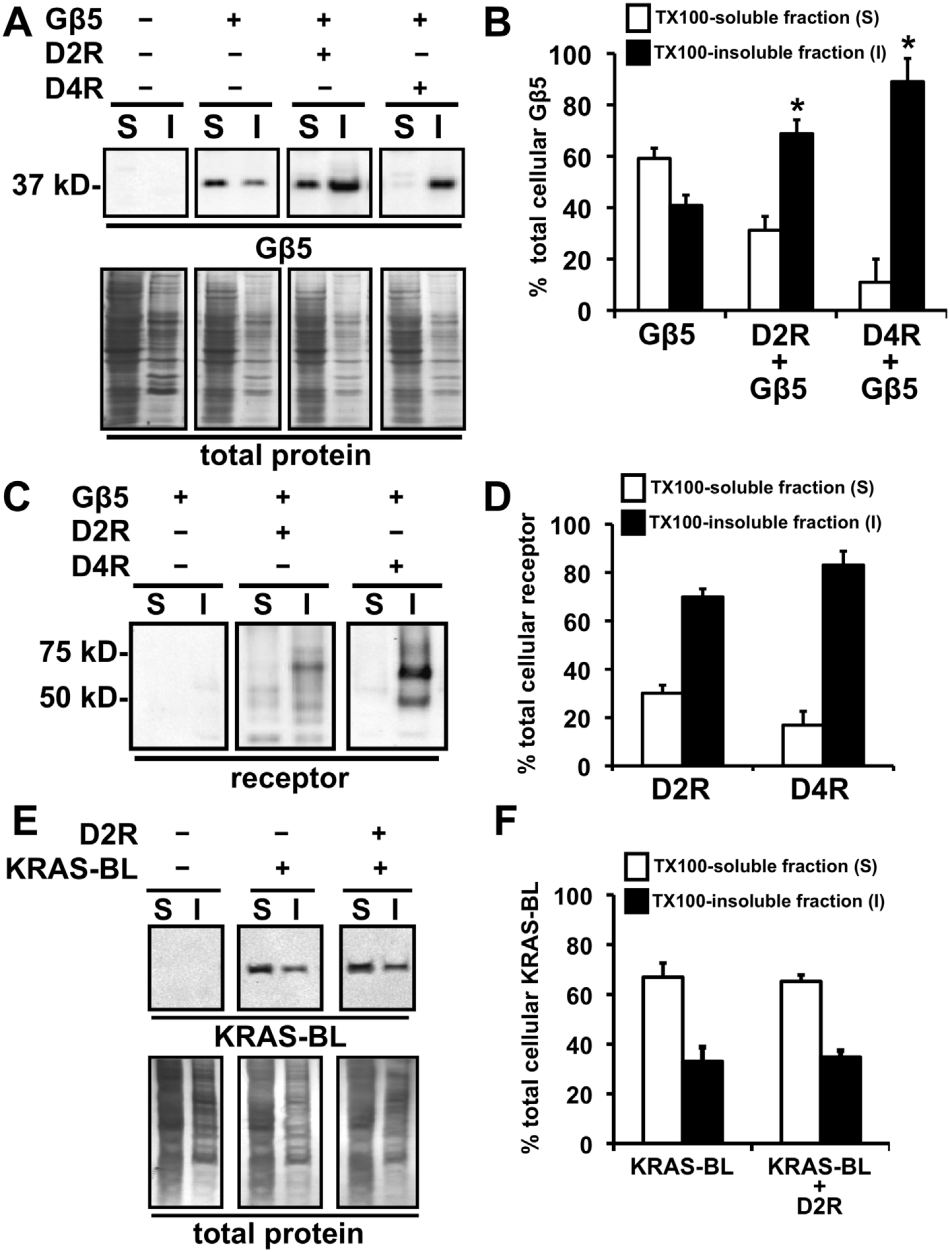
Targeting of Gβ5 to the TX100-insoluble fraction upon coexpression of D2-like dopamine receptors, D2R and D4R. **A.** Representative image of a Western blot depicting the segregation of Gβ5 (upper panels) and total protein (lower panels) into TX100-soluble (S) and insoluble (I) biochemical fractions prepared from HEK293 cells transfected with cDNAs for the indicated proteins. **B.** Quantification of the relative levels of Gβ5 segregating into TX100-soluble (white bars) and TX100-insoluble (black bars) biochemical fractions expressed as percentage of the total cellular Gβ5 signal from the respective cellular samples (mean ± SEM; n = 4, *p<0.01, t-test relative to cells expressing Gβ5 alone. **C.** Representative image of a Western blot depicting the segregation of the respective FLAG-tagged dopamine receptor proteins, D2R and D4R, into TX100-soluble (S) and insoluble (I) biochemical fractions prepared from HEK293 cells transfected with the indicated cDNAs. **D.** Quantification of the relative levels of D2R and D4R segregating into TX100-soluble (white bars) and TX100-insoluble (black bars) biochemical fractions prepared from cell samples indicated in C (mean ± SEM; n = 4). **E.** Representative image of a Western blot depicting the segregation into TX100-soluble (S) and insoluble (I) biochemical fractions of transiently expressed KRAS-BL (upper panels) and total protein (lower panels) in HEK293 cells and effect of transient coexpression of D2R on such segregation. **F.** Quantification of the relative levels of KRAS-BL segregating into TX100-soluble (white bars) and TX100-insoluble (black bars) biochemical fractions (mean ± SEM; n = 4).

One explanation for the increased detergent-resistance of striatal Gβ5 is that D2R, which we have shown is highly resistant to detergent solubilization, is expressed at high concentrations in the striatum compared to the cortex and Gβ5 is then targeted to the detergent-resistant striatal D2R through an interaction with RGS9-2 or other R7 RGS proteins [24]. Therefore, in a control experiment using HEK293 cells, we tested if D2R could enhance the detergent-resistance of Gβ5 independently of exogenously expressed R7 RGS proteins. We found that coexpression of D2R with Gβ5 in HEK293 cells significantly increased the percent of Gβ5 that segregated into the TX100-insoluble cellular fraction (from ∼40% to 70%), even in the absence of exogenously coexpressed R7 RGS protein constructs (Fig. 1A and B). This is a surprising result, because while endogenous expression of R7 RGS proteins in HEK293 cells has been suggested via RNA interference [32], a microarray analysis of mRNA levels of GPCR related signaling proteins expressed in these cells did not detect statistically significant levels of mRNA for any of the R7 RGS proteins [33]. Thus, transiently expressed Gβ5 protein, is likely to vastly exceed the endogenously expressed levels of R7 RGS family members in HEK293 cells.

Coexpression of Gβ5, on the other hand, did not significantly affect the TX100-solubility of D2R protein (the percent of D2R that was soluble when expressed alone was 21.8±4.7, and after Gβ5 coexpression was 30.8±5.4, n = 4, p>0.05, t-test, data not shown). Figures 1C and D illustrate, as reported earlier [24], [25], that the majority (>70%) of coexpressed D2R protein segregates into the TX100-insoluble fraction.

Since D2R coexpression increased the TX100 insolubility of Gβ5 it was important to examine the effect of dopamine pretreatment on the TX100 solubility of both D2R and of Gβ5 coexpressed with D2R. Previously, we had reported that dopamine treatment did not affect the detergent-solubility of D2R [24], [25]. Similarly, we found that dopamine pretreatment (10 µM for 30 min) had no effect on the D2R-mediated recruitment of Gβ5 to the TX100-insoluble cellular fraction, i.e. dopamine pretreatment did not alter the TX100 solubility of Gβ5 when coexpressed with D2R (data not shown).

We report that the closely related D2-like dopamine receptor, D4R, also segregates into the TX100-insoluble cellular fraction and that Gβ5 is similarly retargeted to the TX100-insoluble cellular fraction after D4R coexpression (Fig. 1A, B, C and D).

The dopamine-receptor enhancement of the detergent insolubility of Gβ5 was specific as D2R coexpression did not alter the TX100-solubility of KRAS (Fig. 1E and F), a plasma membrane targeted protein that has been well characterized in the literature to segregate into TX100-soluble cellular fractions [34], [35].

The above phenomenon is specific to dopamine receptors as we have previously reported that coexpression of the mu opioid receptor (MOR) did not affect the detergent-solubility of either component of the R7 RGS protein-Gβ5 complex [24].

The other, more canonical G protein Gβ subunits are intrinsically resistant to detergent solubilization [36], and thus a similar D2R-mediated retargeting of other Gβ subunits, such as Gβ1, to the TX100-insoluble cellular fraction was not observed (Fig. S1A and B).

### D2R coexpression specifically enhances the expression and stability of Gβ5

**Figure 2:**
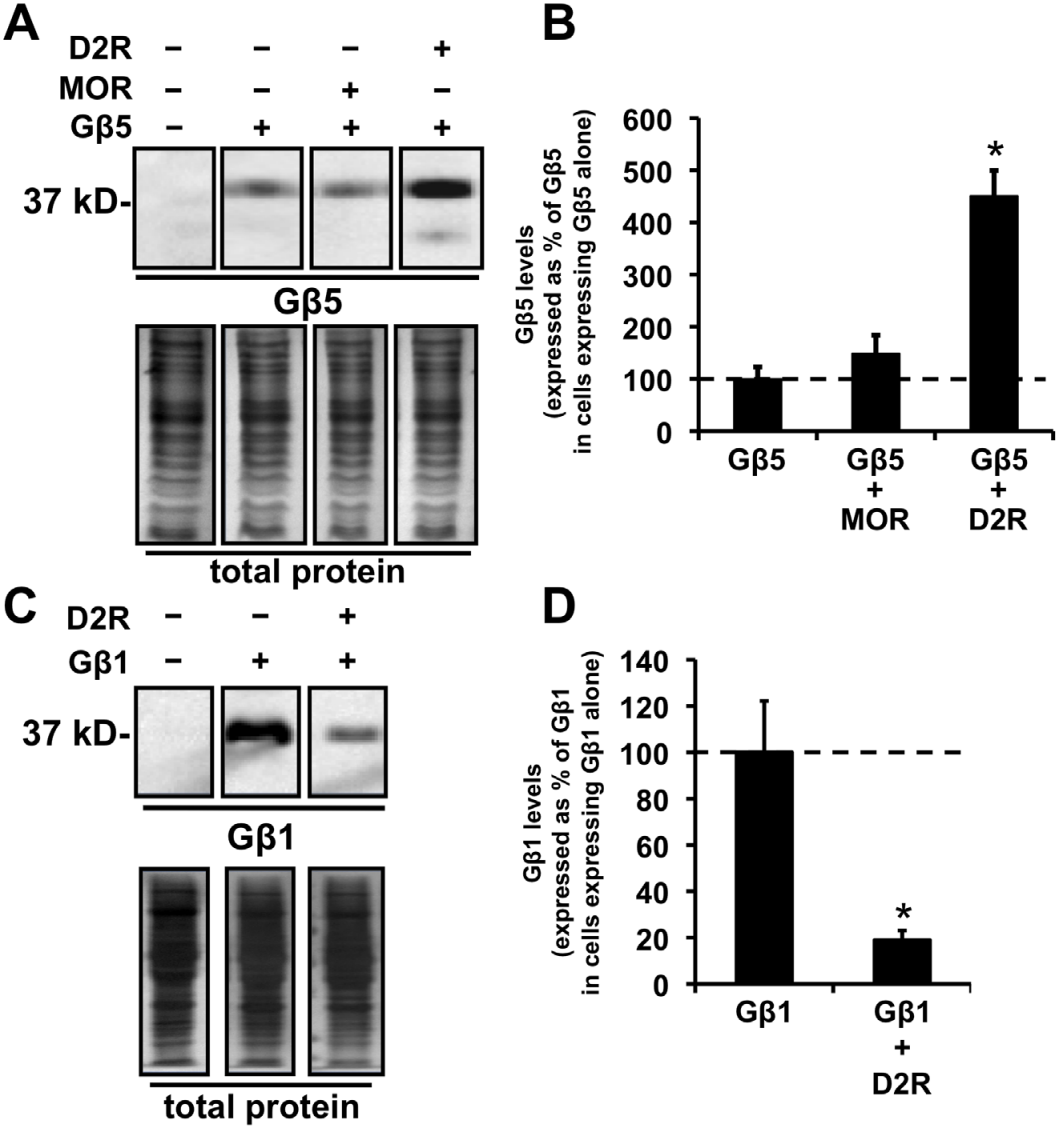
Coexpression of D2R enhances the expression of Gβ5 but not of Gβ1 and coexpression of MOR does not significantly alter expression of Gβ5. **A.** Representative images from a Western blot depicting the relative levels of expression of Gβ5 (upper panels) and total cellular protein (lower panels) in HEK293 cells transfected with cDNA for the indicated proteins. **B.** Quantification of the relative cellular expression levels of Gβ5 after coexpression of MOR or D2R. The Gβ5 protein signal is expressed as a percent of the signal measured in cells expressing Gβ5 alone (mean ± SEM; n = 4, *p<0.05, ANOVA followed by Tukey’s post-hoc test, compared to the levels in cells expressing just Gβ5). **C.** Representative images from a Western blot depicting the relative levels of expression of Gβ1 (upper panels) or total cellular proteins (lower panels) in HEK293 cells transfected with cDNA for the indicated proteins. **D.** Quantification of the relative cellular expression levels of Gβ1 after coexpression of D2R. The Gβ1 protein signal is expressed as a percent of the signal measured in cells expressing Gβ1 alone (mean ± SEM; n = 3, *p<0.05, t-test, compared to the levels in cells expressing just Gβ5).

In addition to translocating Gβ5 to the TX100-insoluble fraction we observed that the coexpression of D2R simultaneously and dramatically increased the cellular expression of Gβ5 protein (Fig. 1A, 2A and B).

The actions of D2R in increasing Gβ5 expression levels were specific. First, coexpression of D2R increased expression levels of Gβ5 by more than 400%, but, in contrast, coexpression of the closely related dopamine receptor, D4R, did not enhance the expression levels of Gβ5. The Gβ5 expression level with D4R coexpressed was only 87.3±24.7% (mean ± SEM, n = 4) of Gβ5 levels in cells expressing Gβ5 alone. Coexpression of another GPCR, the mu opioid receptor (MOR), also did not significantly alter the expression levels of Gβ5 (Fig. 2A and B).

Second, the expression level of the G protein Gβ subunit, Gβ1, was instead, significantly decreased after D2R coexpression (Fig. 2C and D).

**Figure 3:**
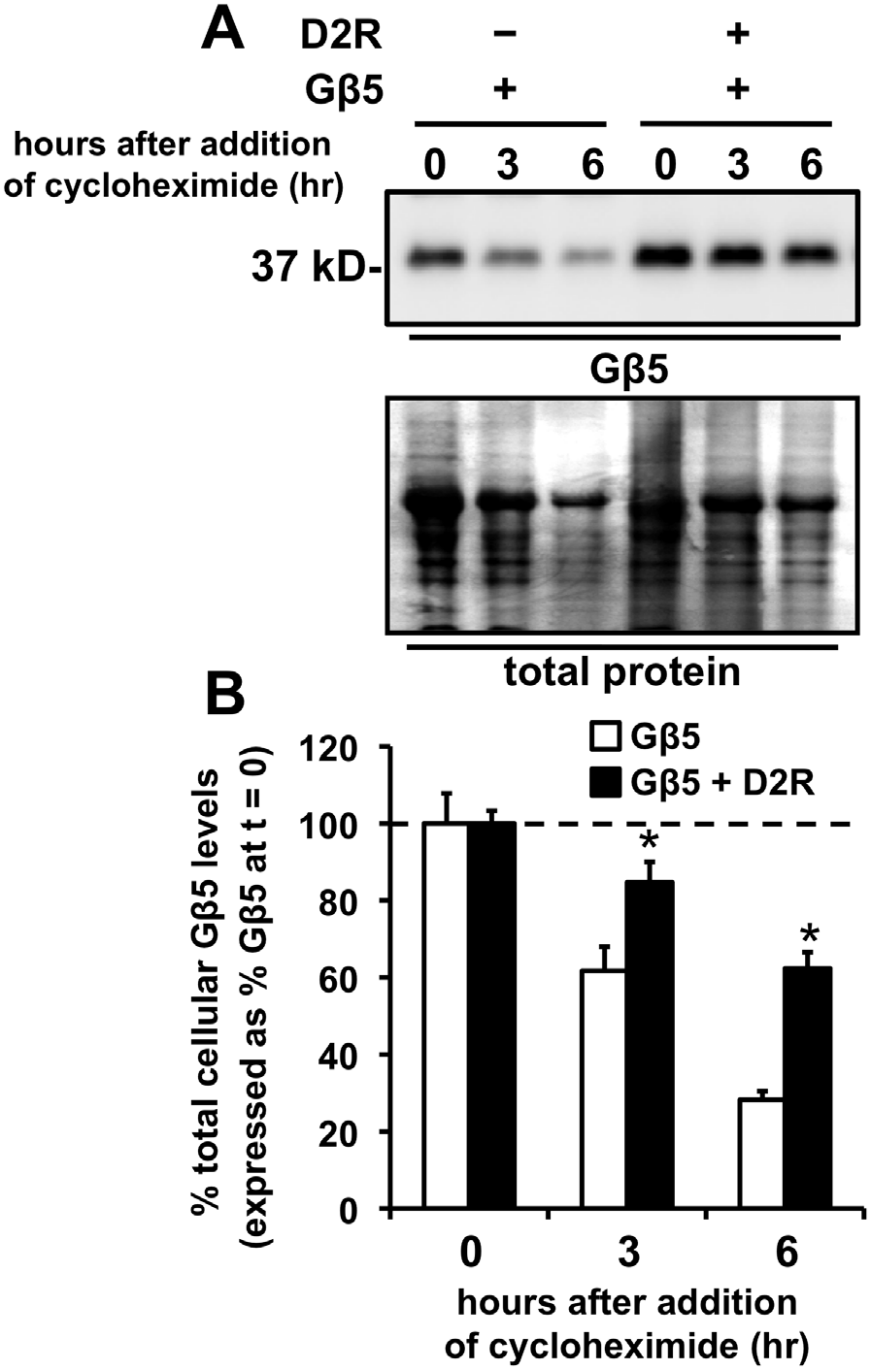
Coexpression of D2R enhances the stability of Gβ5. **A.** Representative image of a Western blot which depicts Gβ5 cellular expression levels levels (upper panel) or total cellular protein (lower panel), from HEK293 cells transiently expressing either Gβ5 alone or Gβ5 coexpressed with D2R, at times, t = 0, 3, or 6 hr after treatment with cycloheximide (100 µM). **B.** Quantification of the reduction in cellular Gβ5 levels after treatment of cells with cycloheximide. The Gβ5 levels at times 3 and 6 hr after cycloheximide treatment are expressed as a percentage of the levels of Gβ5 measured in cells that were not treated with cycloheximide (mean ± SEM; n = 4, *p<0.05, t-test, comparing to the cells that did not coexpress D2R).

To explore if D2R-mediated stabilization of Gβ5 contributed to the enhanced Gβ5 expression observed after D2R expression, we treated HEK293 cells expressing Gβ5 alone, or coexpressing D2R and Gβ5, with cycloheximide, a protein translation/synthesis inhibitor, and the decay of the cellular Gβ5 protein signal after cycloheximide treatment for 3 and 6 hr was monitored by Western blotting. We found that coexpression of D2R significantly decreased the decay of the Gβ5 signal observed at both 3 and 6 hr (Fig. 3A and B). For example, after 6 hr of cycloheximide treatment, the levels of Gβ5 protein in cells expressing Gβ5 alone had decayed to less than 30%, but in cells coexpressing D2R greater than 60% of the original Gβ5 signal remained (Fig. 3). Thus, D2R coexpression significantly inhibited the cellular degradation of Gβ5.

### An in-cell proximity biotinylation assay indicates that the detergent-insoluble D2R is relatively accessible to Gβ5

Previously, we had shown that the detergent-insoluble pool of D2R, which forms the vast majority of the cellular D2R, represents receptor that is micro-compartmentalized in the plasma membrane [25]. The microcompartmentalized D2R is accessible to proteins such as β-arrestin, which has previously been shown to interact with the receptor. However, the microcompartmentalized D2R does not interact readily with other randomly selected plasma membrane-targeted proteins.

One explanation for the redistribution of Gβ5 to the TX100-insoluble cellular fraction after D2R coexpression (Fig. 1A, B, C and D), is that Gβ5 is targeted either directly or indirectly to the TX100-insoluble microcompartmentalized D2R. Hence, we decided to compare the accessibility of the TX100-insoluble pool of cellular D2R to Gβ5 and a randomly selected protein such as KRAS. We could not use traditional coimmunoprecipitation techniques [37] for probing for either direct or indirect physical interactions between the TX100-insoluble D2R and Gβ5 because these techniques first require solubilizing the proteins in non-ionic detergents that preserve protein-protein interactions. Unfortunately, the vast majority of D2R is insoluble in these nonionic detergents. Furthermore, other technologies for probing protein-protein interactions such as fluorescence or bioluminescence resonance energy transfer (FRET or BRET) [38] cannot report if D2R and Gβ5 molecules that specifically segregated into the detergent-insoluble cellular fraction had also interacted in living cells.

**Figure 4:**
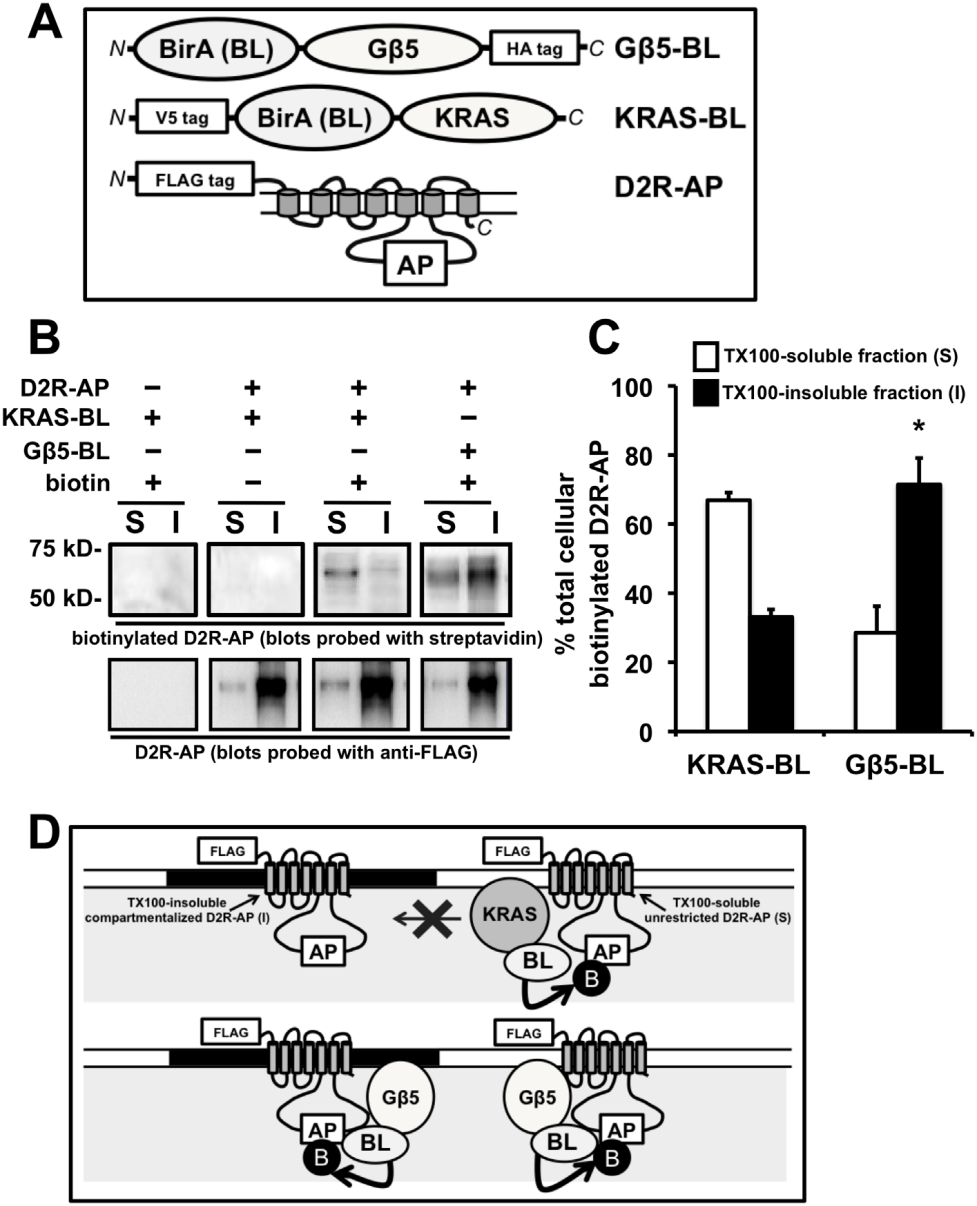
Interactions of Gβ5 with TX100 soluble (S) and insoluble (I) D2R populations as assessed by an in-cell “proximity biotin transfer assay”. **A.** Schematic of the Gβ5-BL construct, a fusion of Gβ5 with BirA, an E. coli derived biotin ligase (BL) enzyme that specifically biotinylates a unique acceptor peptide (AP) sequence (top panel). N and C refer to the N- and C-terminus, respectively, of the fusion protein. Schematic of KRAS-BL, a fusion of the BirA biotin ligase (BL) enzyme with the plasma membrane targeting peptide motif from KRAS (center panel). Schematic of the D2R-AP fusion construct where the AP sequence that is specifically biotinylated by BirA was inserted into a region of the 3rd cytoplasmic loop of FLAG-tagged D2R (bottom panel). **B.** Representative image of a Western blot depicting the segregation, into TX100-soluble (S) and insoluble (I) HEK293 cellular fractions, of the D2R-AP construct that was biotinylated by either co-expressed Gβ5-BL or KRAS-BL (upper panels) or the distribution of the parent D2R-AP protein (lower panels). The left and center panels represent samples prepared from HEK293 cells transiently coexpressing D2R and Gβ5-BL. Samples in the left panel were from cells not treated with biotin (biotin −). Samples depicted in the center panel were from cells transiently coexpressing D2R and KRAS-BL and samples depicted in the right panel were from cells transiently coexpressing D2R and Gβ-BL. Cells were cultured in biotin-depleted medium and biotinylation of the D2R-AP construct by either Gβ5-BL or KRAS-BL was initiated in intact cells by treatment with 10 µM biotin for 2 min (biotin +). Biotinylated D2R-AP segregating into TX100-soluble (S) and insoluble (I) fractions was detected by probing the blots with streptavidin, parent D2R-AP was detected by probing identically loaded blots with FLAG antibody. **C.** Quantification of the percent of the biotinylated D2R-AP segregating into TX100-insoluble (I) and soluble (S) fractions after biotinylation by either KRAS-BL or Gβ5-BL (mean ± SEM; n = 4, *p<0.05, t-test, compared to the segregation after biotinylation by KRAS-BL). **D.** Schematic of an explanation for the data presented in B and C. The majority of the D2R-AP construct is expressed in plasma membrane domains that are TX100-insoluble (black filled-in region of the plasma membrane) but some D2R-AP is also expressed in a TX100-soluble form (white open region of the plasma membrane). Gβ5-BL is able to access, interact with and biotinylate both forms with equal efficacy (lower panel). The interactions of KRAS-BL, on the other hand, are largely restricted to the TX100-soluble form of D2R-AP indicated that it is compartmentalized away from the TX100-insoluble D2R-AP (upper panel).

Thus, to compare the level of interaction of between the TX100-insoluble D2R and Gβ5 or KRAS in living cells, we utilized a novel in-cell proximity biotinylation assay. This assay involves the *E. coli* biotin ligase, BirA [25], [39], which specifically biotinylates a unique “acceptor peptide” sequence, not present in mammalian proteins. An attenuated biotinylation acceptor peptide substrate sequence (denoted here as AP) was inserted into the 3^rd^ cytoplasmic loop of D2R (D2R-AP), while the BirA biotin ligase enzyme (BL) was fused to either Gβ5 (Gβ5-BL) or a peptide motif from KRAS (KRAS-BL) (Fig. 4A). The D2R-AP substrate and the biotin ligase enzyme fusions were co-expressed in HEK293 cells cultured in biotin-depleted medium. Following a brief (2 min) treatment of the intact living cells with biotin, the cells were lysed in cold (4°C) TX100 lysis buffer and separated into TX100-soluble and insoluble fractions. Biotinylation of D2R-AP provides evidence for interactions between the D2R-AP substrate and coexpressed biotin ligase-containing fusions that had occurred in the intact living cell, because these two proteins must come within close proximity (∼2.2 nm) in order for biotinylation to occur.

The use of the technique to evaluate the level of interaction between two proteins in living cells has been previously validated in multiple studies. For example, the rapamycin-induced interaction between the FK506 binding protein (commonly referred to as FKBP) and the FKBP-rapamycin binding protein could be detected by enhanced in-cell biotinylation of an FKBP-AP fusion substrate by an FKBP-rapamycin binding protein-BL fusion [39]. Similarly, we found that the in-cell biotinylation of D2R-AP fusions by a β-arrestin2-BL fusion protein was enhanced by treatment of the cells with dopamine [25].

We had reported earlier that the insertion of the AP-tag into D2R does not greatly affect its detergent solubility [25] and that the vast majority (∼80%) of the D2R-AP construct segregated into the TX100-insoluble cellular fraction. We also showed previously, that when D2R-AP fusion substrates and a wide variety of peptide motifs and cellular proteins fused to the biotin ligase enzyme were coexpressed in HEK293 cells, in almost every case, the majority of the biotinylated D2R-AP substrate segregated into the TX100-soluble fraction [25]. This occurred even though the vast majority of the parent D2R-AP substrate protein localized into the TX100-insoluble fraction [25]. These results indicate that the detergent-resistant D2R, though functional and expressed in the plasma membrane, as we previously showed [25], represents receptor that is compartmentalized from interacting non-specifically with other cellular proteins. On the other hand, the detergent-soluble D2R, which represent a minority of the cellular D2R, likely originates from a more fluid region of the cell membrane and can interact randomly with other cellular proteins according to the fluid mosaic model of Singer and Nicolson [40].

In accordance with the above results, we show that the majority (∼70%) of D2R-AP that was biotinylated by KRAS-BL (which was determined by probing the blots with streptavidin) segregates into the TX100-soluble fraction (Fig. 4B and C) even though the majority (>70%) of the parent D2R-AP protein is found in the TX100-insoluble fraction (Fig. 4B and C). An interpretation of the above results is that the small minority of cellular D2R-AP that is present in the TX100-soluble and hence fluid region of the plasma membrane can interact randomly and be biotinylated by KRAS-BL. The major cellular pool of D2R-AP (i.e. the TX100-insoluble D2R-AP pool) is compartmentalized and the accessibility of KRAS-BL to this pool is significantly inhibited compared to the TX-soluble D2R-AP molecules.

In contrast, we found that the segregation of D2R-AP biotinylated by Gβ5-BL, more closely matched the segregation of the parent D2R-AP protein, with ∼70% of biotinylated D2R-AP segregating into the TX100-insoluble fraction (Fig. 4B and C). In other words Gβ5-BL could equally access both the TX100-insoluble and soluble pools of D2R-AP molecules. These results may be interpreted to suggest that 1) Gβ5, unlike other cellular proteins, efficiently interacts in living cells with D2R molecules that segregates into TX100-insoluble cellular fractions and 2) that D2R segregating into the TX100-resistant cellular fraction (i.e. the majority of the plasma membrane-expressed D2R) is not compartmentalized from Gβ5 as it was from KRAS and many other cellular proteins (Fig. 4D).

### Effect of coexpression of Gβ5 on cellular coupling between D2R and Gαo G proteins

We then tested if the coexpression of Gβ5 could alter the cellular functions of D2R. To test the effects of Gβ5 coexpression on D2R-mediated G protein activation we utilized a bioluminescence resonance energy transfer (BRET) based assay, recently developed by Hollins and colleagues [41]. This assay measures the release of free Gβγ subunits from the activated G protein. The BRET pair that is utilized is the Gβγ dimer tagged with Venus (Gβγ-Venus) and masGRK3ct-NanoLuc [42]. The use of this system to monitor coupling between D2R and associated G proteins has been described in detail in a previously published study [30]. Briefly, the following proteins were coexpressed in HEK293 cells: D2R, the D2R coupled G protein subunit, G protein alpha (o) (Gαo), Gβγ-Venus and masGRK3ct-NanoLuc. Activation of the coexpressed G proteins by dopamine-bound D2R results in the release of the Venus-tagged Gβγ dimers from the activated Gα subunits and interaction with the NanoLuc-tagged masGRK3ct reporter produces the BRET signal. Subsequent application of the D2R antagonist, haloperidol, results in the reversal of activation of D2R-coupled Gαo G proteins and a re-equilibration of free Gβγ-Venus from the Gβγ-Venus-masGRKct-NanoLuc complex to the GDP-bound Gα subunit resulting in the reversal of the BRET signal. No significant dopamine-elicited response was observed in cells not transfected with cDNA for either D2R or Gαo [30] indicating that the BRET signal results from the activation of exogenously expressed Gαo G proteins by D2R.

**Figure 5:**
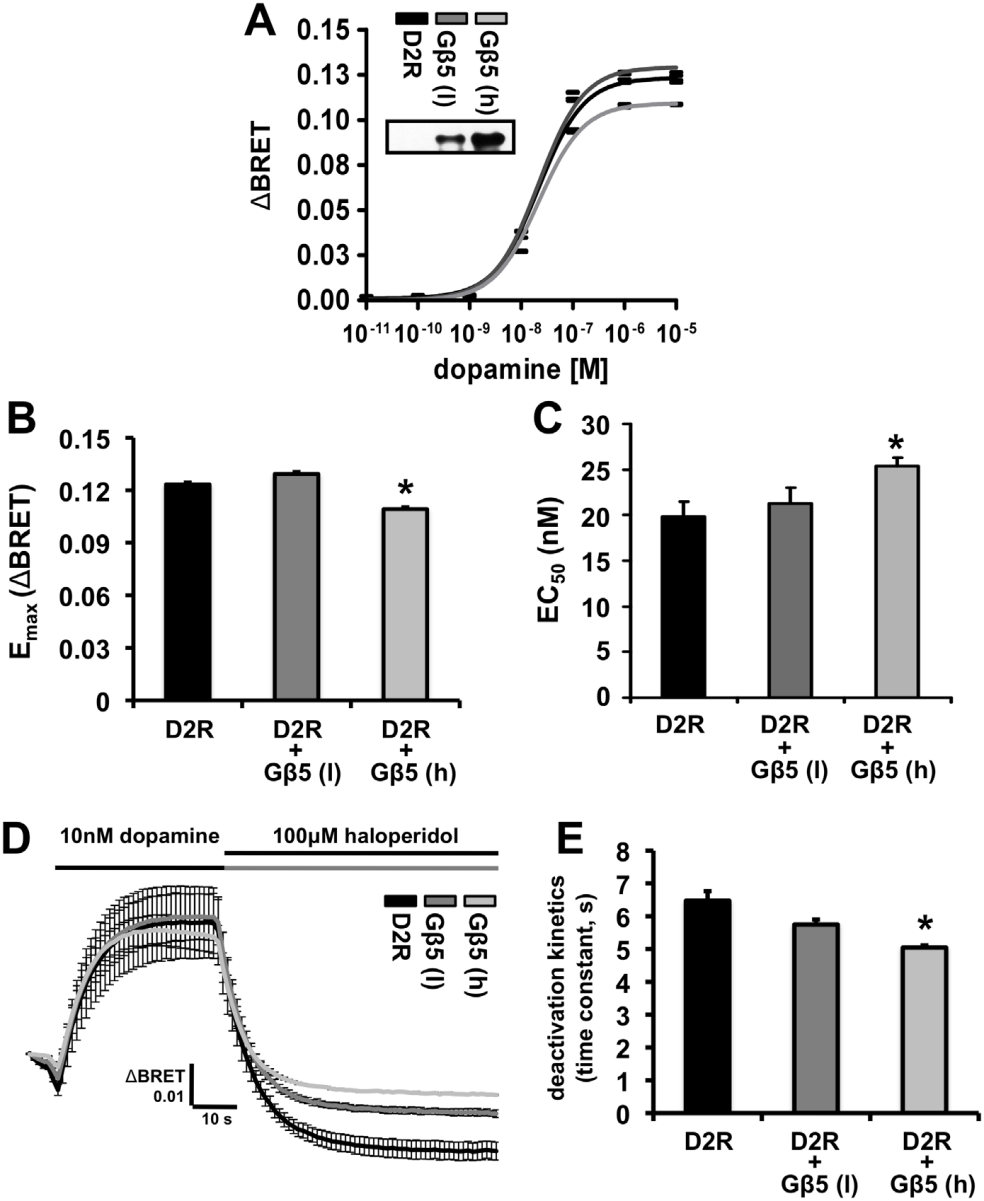
Effect of Gβ5 on dopamine-mediated activation of D2R-coupled G protein signaling as measured by a fast kinetic BRET assay. **A.** Average BRET dose-repsonse curve elicited by the application of 10 pM - 10 µM concentrations of dopamine to cells expressing only D2R (black trace) and cells transiently coexpressing low (dark grey) and high (light grey) levels of Gβ5. D2R stimulation by dopamine application leads to the dissociation of the G protein heterotrimer into Gβγ-Venus and GTP-bound Gαo subunits. Free Gβγ-Venus interacts with masGRK3ct-NanoLuc to produce a BRET signal. The black trace is from HEK293 cells that did not coexpress Gβ5 and the dark and light grey traces are from HEK293 cells transiently coexpressing two different levels (l, for low and h, for high) of Gβ5 (mean ± SEM; n = 4). **B.** Quantification of the maximal amplitude (E_max_) of the BRET signal elicited by the application of dopamine in the cells described above. The E_max_ only significantly differed between D2R and D2R + Gβ5 (h) (*p<0.01, ANOVA followed by Tukey’s post-hoc test). **C.** Quantification of the average EC_50_ derived for the dopamine mediated activation of D2R in the cells described in D. The response at each dose is expressed as a percent of the average maximal response (n = 4). The EC_50_ (in nM) for D2R (black bar), D2R + Gβ5 (l, dark grey bar), and D2R + Gβ5 (h, light grey bar) was 19.8±0.825, 21.3±0.863, and 25.4±0.431 (mean ± SEM), respectively. The EC_50_ only significantly differed between D2R and D2R + Gβ5 (h) (*p<0.01, ANOVA followed by Tukey’s post-hoc test). **D.** Averaged traces (± SEM) of changes in the BRET signal (ΔBRET or the BRET response) over time obtained from HEK293 cells transfected with cDNA for D2R, Gαo, Venus-Gβγ, masGRK3ct-NanoLuc and treated sequentially with dopamine (10 nM) and haloperidol (100 µM). **E.** Quantification of the deactivation kinetics of the dopamine-elicited BRET response after application of the D2R antagonist, haloperidol (100 µM).

Using this assay system we generated dopamine dose-response curves for the D2R-mediated activation of the BRET response in the presence or absence of coexpressed Gβ5. Cells were cotransfected with two concentrations of Gβ5 cDNA: the lower concentration, denoted as Gβ5 (l) in Fig. 5, was the concentration utilized in all of the other experiments described here and a higher concentration, denoted as Gβ5 (h), that produced much greater Gβ5 protein expression levels (> 300%). The transfection of the lower level of Gβ5 cDNA, Gβ5 (l), produced no significant alterations in the maximal dopamine response (E_max_) or the dopamine EC_50_ concentration (Fig. 5, A, B and C). The high Gβ5 concentration, Gβ5 (h), produced a small but significant increase in the dopamine EC_50_ and a corresponding small but significant decrease in the E_max_.

We then examined the effects of Gβ5 coexpression on the deactivation kinetics of D2R-Gαo G proteins signaling where the dopamine signal obtained by perfusing cells with 10 nM dopamine was reversed by the application of 100 µM haloperidol. At the lower level of Gβ5 expression, Gβ5 (l), no significant effect was observed on the deactivation kinetics (Fig. 5D and E). When Gβ5 was expressed at the much higher level, Gβ5 (h), a small but significant acceleration of the deactivation kinetics was detected.

### Coexpression of Gβ5, but not Gβ1, inhibits dopamine-induced internalization of D2R, and Gβ5 coexpression does not affect agonist-induced internalization of MOR

**Figure 6:**
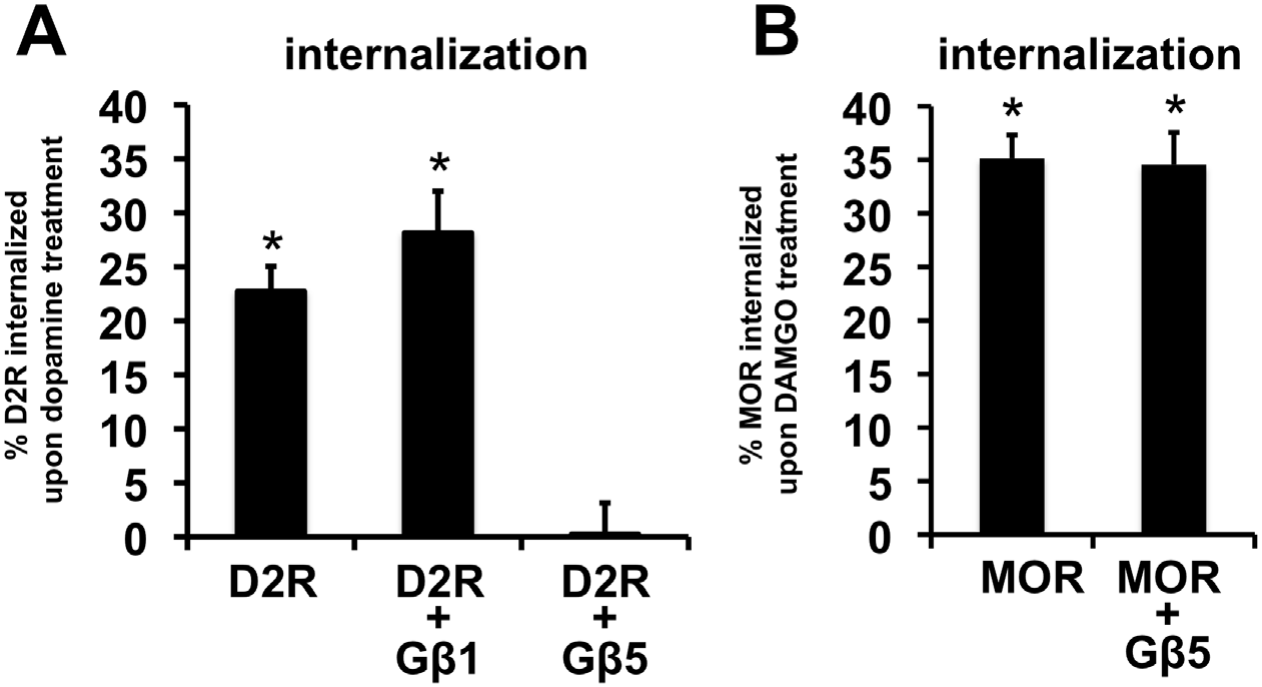
Effects of Gβ5 and Gβ1 coexpression on agonist-induced receptor internalization. **A.** Quantification of the relative levels of transiently expressed cell surface D2R in HEK293 cells expressing D2R alone, D2R and Gβ1 or D2R and Gβ5, after treatment of with dopamine (50 µM for 45 min). The bars represent the percentage decrease in cell surface D2R in each transfection condition compared to corresponding cells that were not treated with dopamine (mean ± SEM; n = 8, *p<0.01, t-test, compared to cell surface D2R signal from corresponding cells not treated with dopamine). **B.** Quantification of the relative levels of transiently expressed cell surface MOR in HEK293 cells expressing MOR alone and MOR with Gβ5, after treatment of DAMGO (50 µM for 45 min). The bars represent the percentage decrease in cell surface MOR in each transfection condition compared to corresponding cells that were not treated with DAMGO (mean ± SEM; n = 11–12, *p<0.001, t-test, compared to cell surface MOR signal from corresponding cells not treated with DAMGO).

To quantify receptor internalization we measured the amount of receptor at the surface of HEK293 cells both before and after agonist treatment through a modification of a previously described enzyme-linked immunosorbent assay (ELISA)-based protocol (see Materials and Methods) [43]. Treatment of cells transiently expressing D2R or MOR for 45 min with the respective receptor agonists, dopamine or DAMGO, significantly reduced cell surface levels of the respective receptors (Fig. 6A and B). Coexpression of Gβ1 had no effect on the loss of cell surface D2R produced by dopamine treatment. In contrast, coexpression of Gβ5 completely blocked the dopamine-induced internalization of D2R (Fig. 6C) but had no effect on DAMGO-induced internalization of MOR (Fig. 6B).

The Gβ5-mediated inhibition of dopamine-induced D2R internalization was not an artifact of alterations in surface receptor levels, as coexpression of Gβ5 had no significant effect on the cell *surface levels* of D2R or MOR (Fig. S2A and B).

### Coexpresson of Gβ5 does not affect the dopamine-dependent recruitment of arrestin to D2R

**Figure 7:**
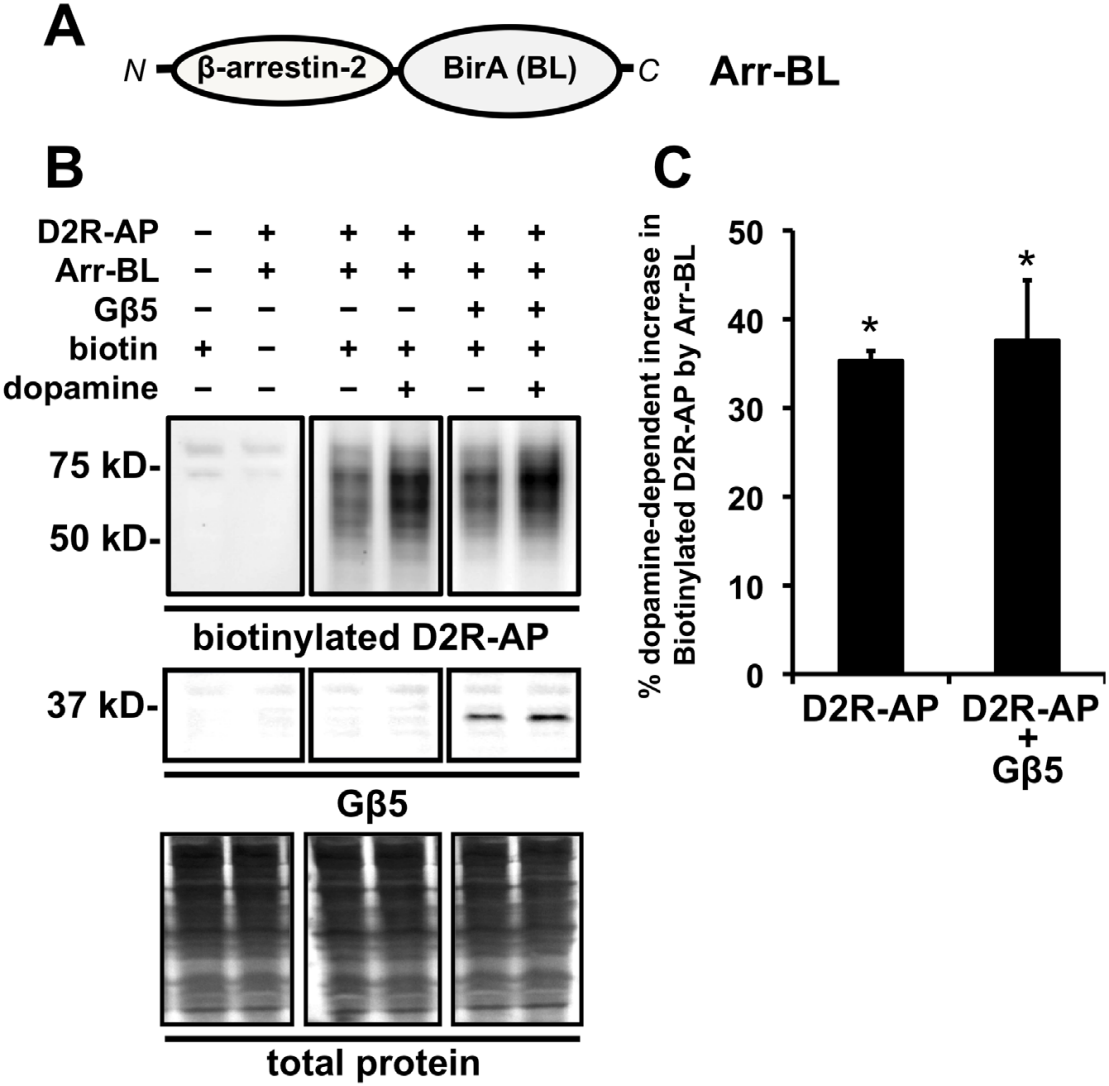
Effect of Gβ5 coexpression on arrestin recruitment to D2R-AP upon dopamine treatment as assessed by an in-cell “proximity biotin transfer assay”. **A.** Schematic of the Arr-BL construct, a fusion of arrestin and BirA, an *E. coli* derived biotin ligase (BL) enzyme that specifically biotinylates a unique acceptor peptide (AP) sequence. N and C refer to the N- and C-terminus, respectively, of the fusion protein. **B.** Representative images from a Western blot depicting total cellular biotinylated D2R-AP by coexpressed Arr-BL (top panels) and coexpressed Gβ5 (bottom panels). The top left panel represents samples prepared from cells that were untransfected with either D2R-AP (−) or Arr-BL (−) and treated with 10 µM biotin for 2 min (biotin +, left) or samples that were transfected with both D2R-AP (+) and Arr-BL (+) and not treated with biotin (biotin −, right). The top center panel represents samples prepared from cells which were transfected with both D2R-AP (+) and Arr-BL (+) and treated with vehicle (dopamine −, left) or 10 µM dopamine for 30 min (dopamine +, right) and treated with biotin. The top right panel represents samples prepared from cells which were transfected with both D2R-AP (+), Arr-BL (+), and Gβ5 (+) and treated with vehicle (dopamine −, left) or 10 µM dopamine for 30 minutes (dopamine +, right) and treated with biotin (+).D2R-AP biotinylated by Arr-BL was detected by probing the blots with streptavidin. The middle panels represent corresponding western blots from samples in the upper panel probed for Gβ5. The bottom panels represent corresponding western blots from samples in the upper panel stained for total protein. **C.** Quantification of the relative levels of D2R-AP biotinylated by Arr-BL in response to dopamine treatment (10 µM for 30 min) in cells expressing only D2R-AP and Arr-BL or cells expressing D2R-AP, Arr-BL, and Gβ5. The biotinylated D2R-AP signal is expressed as a percentage of biotinylated D2R-AP signal from cells that were not treated with dopamine (mean ± SEM; n = 4, *p<0.05, t-test, compared to biotinyalted D2R-AP signal from corresponding cells not treated with dopamine).

The canonical model for the agonist-induced internalization of many GPCRs involves the recruitment, to the agonist-bound GPCR, of β-arrestins, which then serve to physically bridge the receptor to the cellular endocytotic machinery [44]. To determine whether Gβ5 inhibited dopamine-induced D2R internalization by suppressing recruitment of β-arrestin we used the in-cell proximity biotin-transfer assay to evaluate the actions of Gβ5 on this process. In this assay, D2R-AP and a fusion construct of β-arrestin2 and the *E. coli* biotin ligase BirA (Arr-BL) (Fig. 7A) are transiently expressed in HEK293 cells and dopamine treatment (10 µM for 30 min) significantly enhances the Arr-BL -mediated biotinylation of D2R-AP [25] (Fig. 7B and C). However, coexpression of Gβ5 had no effect on D2R-AP biotinylation suggesting that Gβ5 did not inhibit recruitment of β-arrestin to D2R.

The failure to observe any Gβ5-mediated inhibition of β-arrestin recruitment to D2R was not due to any limitation of the proximity biotinylation assay. Previous studies have established that it is protein kinase C (PKC)-mediated phosphorylation of D2R and not GRK phosphorylation that is required for dopamine-induced recruitment of β-arrestin to D2R [45]–[48]. We therefore performed a validation experiment by treating cells with staurosporine, a potent PKC and serine and threonine kinase inhibitor [49], and observed that staurosporine completely blocked the dopamine-dependent, Arr-BL -mediated enhancement of D2R-AP biotinylation. Additionally, we found that transient coexpression of β-arrestin-2 was able to partially block (∼50%) the dopamine-dependent biotinylation of D2R-AP by Arr-BL as would be expected, since the β-arrestin-2 should compete with Arr-BL for binding to D2R-AP (Fig. S3A and B).

## Discussion

### D2R-Gβ5 co-compartmentalization can occur independently of R7 RGS proteins

Several lines of investigations have led to the supposition that Gβ5 is found expressed only as a heterodimer with R7 RGS family proteins [5], [13], . However, none of these experiments have excluded the possibility that alternative proteins could contribute to stabilizing Gβ5 expression in some tissues. The data presented here, using HEK293 cells, suggest the novel hypothesis that D2R can interact with and stabilize the Gβ5 protein, independently of R7 RGS proteins.

It has been shown that the complexes of Gβ5 and R7 RGS proteins can target to D2R and other GPCRs, and these interactions are mediated through the domains present in R7 RGS proteins such as the DEP domain [19]–[22]. However, the D2R-Gβ5 interactions reported here likely occur independently of R7 RGS protein for the reasons discussed below.

First, Atwood and colleagues have conducted a microarray screen for G protein coupled receptors (GPCR) and related signaling molecules that are endogenously expressed in HEK293 cells and found that the detected levels of transcripts for R7 RGS family members were below levels deemed to be statistically significant [33]. Thus, stoichiometrically, the levels of Gβ5 transiently expressed in HEK293 cells likely far exceed the levels of any endogenously expressed R7 RGS proteins.

Furthermore, we have shown that endogenous expression of Gβ5 is not detected in HEK293 cells [24], and multiple groups, including ours, have shown that, in heterologous expression systems, the observed GTPase accelerating protein (GAP) function of R7 RGS proteins is dramatically enhanced (over 3 fold) by transient coexpression of Gβ5 [15], [30], [50], [51]. The Gβ5 enhancement of GAP function likely occurs through multiple mechanisms including 1) direct conformational alteration of R7 RGS proteins that promote GAP function, 2) through an increase in expression of R7 RGS proteins and 3) by facilitating the interaction of R7 RGS proteins with membrane anchors [50], [52]. Thus, if a significant proportion of the exogenously expressed Gβ5 associates with endogenously expressed R7 RGS proteins it is expected that the formation of such a complex should substantially accelerate the deactivation kinetics of D2R-G protein coupling. However, only a slight acceleration was observed and only when Gβ5 was expressed at a higher level than in the other experiments used to assess interaction with D2R (Fig. 5D and E).

We have previously reported that when R7 RGS proteins, such as RGS9-2, and Gβ5 are transiently expressed in HEK293 cells, D2R co-expression does not significantly alter protein expression levels of either the R7 RGS protein or Gβ5 [24]. In other words, when Gβ5 is present in a complex with R7 RGS proteins, D2R coexpression does not enhance or stabilize Gβ5 protein expression. However, here we have reported that D2R coexpression can dramatically enhance levels of transiently coexpressed Gβ5 protein (Fig. 1A, 2A and B, and 3A), indicating that Gβ5 is not in a complex with endogenously expressed R7 RGS proteins.

Thus, our data suggest that, in HEK293 cells, D2R co-compartmentalizes with Gβ5 in a detergent insoluble biochemical fraction, and in a manner that is independent of R7 RGS proteins. From our data, it is not clear if D2R is interacting with the Gβ5 monomer or with a complex of Gβ5 with other cellular proteins such a G protein Gγ subunits.

### D2R-Gβ5 co-compartmentalization has direct cellular consequences

We found that the co-compartmentalization 1) stabilized and enhanced Gβ5 expression and 2) inhibited dopamine-induced D2R internalization.

It is interesting to note that while the coexpression of both D2R and the closely related dopamine receptor, D4R, enhanced the TX100 insolubility of Gβ5, it was only D2R coexpression that enhanced the protein expression levels of Gβ5 (Fig. 1). Thus, D2R and D4R interact differently with Gβ5 and the evaluation of effects of coexpression of D2R-D4R chimeric constructs on Gβ5 expression may help to define the critical D2R epitopes that help to stabilize Gβ5 in a future study.

Gβ5 at expression levels which strongly inhibited dopamine-induced D2R internalization had no significant effect on D2R-G protein coupling (Fig. 5A, B and C and 6A). It may be then inferred that Gβ5 does not strongly modulate D2R epitopes that are important for activating coupled Gα G proteins but can interfere with D2R interactions that are necessary for internalizing the receptor.

This biased action of Gβ5 in altering D2R cellular functions is particularly interesting. It is now apparent that endogenous agonists may stabilize multiple receptor conformations and the agonist-bound receptor conformation that promotes G protein activation may be different from the conformation that allow for agonist-induced internalization of the receptor [53], [54]. In fact, biased synthetic D2R agonists have been developed that activate non-canonical G protein-independent cellular signals but do not promote D2R-elicited G protein signals [55]. However, we believe that this is the first report of a GPCR-interacting cellular protein that modulates the receptor to abolish agonist-induced internalization but does not affect D2R-G protein coupling. The abolition of dopamine-induced D2R internalization by Gβ5 was not through suppression of D2R interactions with β-arrestin, as Gβ5 did not alter dopamine-induced recruitment of β-arrestin to D2R (7B and C). Gβ5 had no effect on MOR internalization indicating that the prevention of D2R-internalization by Gβ5 likely occurs through a specific targeting of Gβ5 to D2R and is not a consequence of non-specific disruption of the cellular internalization machinery.

A large number of studies have indicated that dopamine-induced internalization of D2R in HEK293 cells is mediated via β-arrestin [45], [47], [48]. This raises the question: how is it possible for Gβ5 to strongly block D2R internalization but have no effect on the dopamine-mediated recruitment of β-arrestin to D2R? One model that may be suggested as an explanation is that internalization of D2R requires one or more bridges between D2R and the cellular internalization machinery, that are in addition to that made through β-arrestin. Gβ5 expression disrupts one or more of these additional connections.

The expression of D2R in detergent-insoluble plasma membrane microcompartments [25] and the targeting of Gβ5 to these microcompartments did not require dopamine pretreatment, indicating that Gβ5 is preassembled in a manner that allows Gβ5 to specifically edit a subset of the actions of dopamine at D2R.

### D2R-Gβ5 co-comparmentalization is not caused by non-specific aggregation of the two proteins

Coexpression of Gβ5 did not alter either the cell surface levels of D2R, the fraction of D2R expressed at the cell surface or the amplitude of D2R-G protein coupling, but clearly inhibited dopamine-induced D2R internalization. These observations indicate that the co-compartmentalization with D2R and stabilization of Gβ5 were not caused by non-specific aggregation of the two proteins.

The majority of the D4-dopamine receptor, which is a member of the D2-like dopamine receptor family, also segregates into detergent-resistant cellular fractions and recruits Gβ5 to the same biochemical fraction. However, these interactions are unique and do not extend to other cell-expressed GPCRs such as mu opioid receptors (MOR), the vast majority of which are readily solubilized in non-ionic detergents [24]. In addition, D2R coexpression does not significantly alter the detergent-solubility of Gβ1 (Fig. S1) or enhance cellular Gβ1 expression levels (Fig. 2C and D).

Here we have provided evidence for a novel and specific feature of Gβ5 that is significant because it suggests that Gβ5 can specifically modulate an important GPCR, D2R, to prevent dopamine-induced D2R internalization without inhibiting G proteins activation. Moreover this action of Gβ5 appears to occur independently R7 RGS proteins.

It is thought that Gβ5 exist in cells as an obligate heterodimer with R7 RGS proteins [5], [13], but such a postulate has not been proven. Our data suggests that in some cells, Gβ5 may be stabilized by protein partners other than R7 RGS proteins, such as D2R. While the expression of both R7 RGS proteins and Gβ5 is thought to be broadly localized to neural, neuroendocrine and other excitable tissues such as heart muscle [5], [6], [13], it is not proven that R7 RGS proteins are coexpressed in all native cells that express Gβ5. Therefore, in some neurons, D2R and Gβ5 may be expressed together, but in the absence of R7 RGS proteins. Furthermore, even if R7 RGS proteins are present in all cells that express Gβ5, in some of these cells the relative R7 RGS protein expression levels may not be high enough to ensure that all of the expressed Gβ5 is bound to the R7 RGS proteins in an obligate manner.

Nevertheless, these experiments were performed in HEK293 cells where concentrations of both D2R and Gβ5 are likely to be higher than that found in native tissue. Hence, definitive *in vivo* evidence for the above suppositions will require further investigations, such as the examination of Gβ5 levels in D2R-expressing cells in mice where all four R7 RGS protein genes are knocked out.

## Materials and Methods

### Chemicals

All chemicals and reagents were purchased from Sigma-Aldrich, Fisher Scientific or from suppliers that have been specifically identified below.

### Cell Culture and Transfection

Human embryonic kidney cells (HEK293, American Type Culture Collection) were grown in Dulbecco's Modified Eagle Medium supplemented with 10% v/v fetal bovine serum, penicillin (100 units/ml) and streptomycin sulfate (100 µg/ml). Mammalian expression plasmids containing the appropriate complementary DNA (cDNA) constructs were transiently transfected using lipofectamine transfection reagent (LTX, Life Technologies) according to the manufacturer’s instructions. Total transfected DNA was maintained between groups by co-transfecting empty plasmid vector pcDNA 3.1+ (Life Technologies).

### cDNA Constructs

All plasmid constructs utilized below were created using standard techniques in molecular biology. The N-terminal FLAG-epitope (FLAG) tagged version of the long form of the human D2-dopamine receptor (D2R) [26], the N-terminal FLAG-tagged D4-dopamine receptor (D4R) [27], the Gβ5 short isoform construct [15], the FLAG-tagged D2R construct with the biotin ligase acceptor peptide insertion into the 3^rd^ cytoplasmic loop (D2R-AP) [25], the *E. coli* BirA biotin-ligase fusion construct with the membrane targeting domain of KRAS (KRAS-BL, and the β-arrestin-2 biotin ligase fusion construct (Arr-BL) [25] have previously been described. The D2R-AP construct consists of the FLAG-tagged D2R into which an attenuated acceptor peptide sequence (GLNDIFEAQKIE) is inserted between amino acids at position 305 and 306 in the 3rd cytoplasmic loop. KRAS-BL consisted of the following peptide sequences in order from the N to the C-terminus: the V5 epitope-tag (V5), the BirA *E. coli* biotin ligase enzyme (BL), a GSGSG linker and a membrane targeting peptide sequence (KKKKKKSKTKCVIM) from the protein KRAS. Arr-BL consisted of the following peptide sequences in order from the N to the C terminus, β-arrestin-2, a GSGSG linker, and the BirA *E. coli* biotin ligase enzyme (BL). The N-terminal FLAG-tagged human G protein Gβ1subunit was obtained from the Missouri S&T cDNA Resource Center (catalog #: GNB010FN00). The G protein beta 5-biotin ligase fusion (Gβ5-BL) was created by attaching the BirA biotin ligase enzyme to the N-terminus of the full-length Gβ5 short isoform via a two amino acid linker. Thus, the fusion protein in order from N to C terminus consists of BirA, an Arg-Tyr linker, the Gβ5 short isoform, and a V5 epitope tag. The cDNA for the *E. coli* biotin ligase, BirA (BL), was provided by Dr. Alice Ting (Massachusetts Institute of Technology) and was amplified by PCR. Diagrams of these constructs are provided in Fig. 4A and 7A.

### Triton X-100 Biochemical Fractionation of Proteins

The method for the Triton X-100 (TX100) biochemical fractionation of proteins has been adapted from our previous publication [24]. Briefly, 48 hr post transfection cells were lysed in TX100 lysis buffer (phosphate-buffered saline, in mM: 137 NaCl, 2.7 KCl, 10 Na_2_HPO_4_, 2 KH_2_PO_4_, pH 7.4 (PBS) containing 2% v/v of the non-ionic detergent, Triton X-100) and a 1× concentration of SigmaFast Protease inhibitor (made according to manufacturer’s instructions, Sigma-Aldrich) for 1 hr at 4°C. The samples were centrifuged (10,000 g, 10 min at 4°C) to pellet the TX100-insoluble proteins. Supernatant proteins (i.e. the TX100-soluble fraction) were precipitated by the addition of trichloroacetic acid (TCA, final concentration 10% v/v). Supernatant proteins were washed 3× with ice-cold 95% v/v acetone (4°C). Both the TX100-soluble and the insoluble proteins were re-suspended in equal volumes of SDS sample buffer (2% w/v SDS, 0.01% w/v bromophenol blue, 8 M urea, 20 mM dithiothreitol, 50 mM Tris-HCl, pH 6.8). Samples were sonicated 25× for approximately 0.5 s at a power setting of 10 for ∼0.5 s to reduce sample viscosity prior to loading using a sonicator (XL-2000, qSonica). Equal volumes of the samples were then resolved by sodium dodecyl sulfate polyacrylamide gel electrophoresis (SDS-PAGE) and the relative levels of protein expression were then compared by Western blotting.

### Protein Degredation Assay

To determine the effect of D2R on the rate of degradation of Gβ5 we used cycloheximide, a translational inhibitor, to block protein synthesis, and then measured the amount of Gβ5 present in cells at 3 and 6 hr after the addition of cycloheximide. 2.5×10^5^ HEK293 cells were transfected with appropriate cDNA plasmids containing Gβ5 with or without D2R in a 24 well-plate. At 48 and 51 hr post-transfection selected wells were treated with 100 µM cycloheximide (time = 6 and 3 hr cycloheximide treatment, respectively). After incubation for 3 hr (54 hr post-transfection) all cell samples were harvested in media from multi-well plates using a micropipetter. Cells were spun down for 5 minutes at 300×g using a benchtop centrifuge and carefully washed 3× with cold (4°C) PBS. Washed cells were then lysed by sonication on ice after being resuspended in equivalent volumes of SDS sample buffer. Protein samples were then incubated for 15 min at 65 °C resolved by SDS-PAGE.

### Biotinylation of D2R-AP by Biotin Ligase Fusion Proteins

We utilized an in cell biotin transfer assay to detect whether or not Gβ5 interacted with the detergentinsoluble form of D2R. The plasmids containing cDNAs for D2R-AP, Gβ5-BL or KRAS-BL as described in Figure 4A were transfected into HEK293 cells in biotin-free media. 48 hr post-transfection, 10 µM biotin was added to the media. After 2 min of incubation at 25°C, the cells were washed 3× using ice-cold (4°C) 1×PBS. The cells were then vigorously resuspended in ice-cold 2% v/v TX100 lysis buffer and incubated for 1 hr at 4°C, with vortexing every 15 min. Soluble and insoluble proteins were then harvested using the method as described in the “Triton X-100 Biochemical Fractionation of Proteins” section.

We also used the biotinylation assay to detect recruitment of β-arrestin 2 to D2R after stimulation by dopamine. The plasmids for D2R-AP and Arr-BL with or without Gβ5 (Fig. 7A) were transfected into HEK293 cells growing in biotin-free media. 48 hours post-transfection, cells were treated with 10 µM dopamine or vehicle for 30 min. Subsequently, the media containing dopamine was removed, the cells were washed 2× with cold (4°C) PBS, and then were treated with 10 µM biotin in 1×PBS for 2 minutes. Cells were then washed 3× with ice-cold PBS and then lysed in SDS sample buffer.

Samples were then resolved by SDS PAGE. The proteins were then transferred to methanol-wetted polyvinylidene fluoride (PVDF) membranes and probed with streptavidin conjugated to horseradish peroxidase (HRP). These procedures and reagents are described in the “Western Blotting” section below.

### Western Blotting

Proteins were resolved by gel electrophoresis and then transferred to methanol-wetted PVDF membranes by Western blotting apparatus (IBI Scientfic) using electrophoresis buffer (25 mM Tris base, 192 mM glycine, 20% v/v methanol, pH ∼8.3). For antibody-based detection, PVDF membranes (Immobilon-FL, EMD Millipore) were blocked by incubation with 5% w/v nonfat dry milk reconstituted in PBS (1 hr at 20°C). For the detection of biotinylated proteins, membranes were blocked by incubation in 3% w/v bovine serum albumin in PBS (1 hr at 20°C). Protein loading controls that are shown are from identically loaded gels, which were transferred to PVDF membranes, stained with 0.01% w/v coomassie blue in 1% v/v acetic acid (5 min at 20°C) and subsequently destained in 50% methanol 5% acetic acid (15 min at 20°C).

V5 epitope-tagged proteins (KRAS-BL and Gβ5-BL) were detected by incubating blots with an HRP-conjugated anti-V5 antibody (Invitrogen/Life Technologies, 1∶5000 in 5% w/v nonfat milk in PBS). FLAG epitope-tagged protein bands (D2R, D4R, and MOR) were detected by incubating blots with an HRP-conjugated mouse monoclonal anti-FLAG M2 antibody (Sigma-Aldrich, 1∶5000 dilution in 5% w/v nonfat milk in 1×PBS). Biotinylated protein (D2R-AP) bands were detected by incubating blots with HRP-conjugated streptavidin (1∶10,000 in 1×PBS containing 3% w/v BSA). Gβ5 was detected by incubating blots with rabbit polyclonal antibody CT215 (1∶5000 in PBS containing 5% w/v nonfat milk) [28], [29]. After incubation with the primary antibodies or HRP-conjugated streptavidin the blots were washed 3× in PBS containing 0.1% v/v tween-20 (PBS-T). If the primary antibody was not directly conjugated to HRP the membrane was then incubated with appropriate HRP-conjugated secondary antibodies (Jackson ImmunoResearch, Inc.) and washed 4× in PBS.

Chemiluminescent signals produced by the HRP enzyme were obtained using Supersignal West Femto substrate and detected using a Chemidoc XRS Molecular Imager (Bio-Rad Laboratories). To directly compare the signals from the TX100-soluble and insoluble fractions of a cell sample, the proteins from these fractions were loaded onto the same SDS-PAGE gel and transferred to a single immunoblot. Protein samples were serially diluted and the signals quantified to ensure that the concentrations used for experiments was in the linear range of the signal-protein function.

### Fast Kinetic BRET Assay

The agonist effects of dopamine on G protein signaling in cells expressing D2R was measured using a fast kinetic bioluminescence resonance energy transfer (BRET) assay. BRET was measured between a Gβγ binding peptide motif from the protein GRK3 fused to a newly engineered luciferase variant, NanoLuc (masGRK3ct-NanoLuc) and Gβ1γ2-Venus in living cells as previously described [30]. BRET measurements were made at room temperature using a microplate reader (POLARstar Omega, BMG Labtech) equipped with two emission photomultiplier tubes, with a maximum of 50 milliseconds resolution. The BRET ratio is calculated by dividing the light emitted by Gβ1γ2-Venus (535 nm) over the light emitted by masGRK3ct-NanoLuc (475 nm). The average baseline value recorded prior to agonist stimulation was subtracted from BRET ratio values, and the resulting difference (ΔBRET) was obtained. The time constants for signal deactivation were derived from single exponential fits of the deactivation curve following application of 100 µM haloperidol. Kinetic analysis and curve fitting were performed using pCLAMP 6 software (Molecular Devices). The average EC_50_ and E_max_ values were derived from separate sigmoidal dose-response curves generated by the standard three-parameter logisitic equation using GraphPad Prism 6.0 (GraphPad Software, Inc.).

### Receptor Internalization Assay

To determine the effect of overexpression of Gβ subunits (Gβ1 or Gβ5) on receptor internalization we used an ELISA-based assay to determine the amount of receptor present at the plasma membrane after the application of dopamine. Day 1, 5×10^4^ HEK293 cells were transfected with appropriate cDNA plasmids containing D2R with or without Gβ1 or Gβ5 or MOR with or without Gβ5, in a 96-well plate. 48 hours post-transfection cells were treated with a saturating concentration (50 µM) of dopamine in the case of D2R or [D-Ala(2), N-Me-Phe(4), Gly(5)-ol]-enkephalin (DAMGO) in the case of MOR for 45 minutes. The media was then aspirated, and cells were gently washed 3× with cold (4°C) PBS. Cells were then fixed with 4% v/v formaldehyde in PBS, and then washed 3× with PBS. Wells were blocked for 30 minutes with 5% nonfat milk dissolved in PBS. Surface receptor was then probed for using HRP-conjugated mouse monoclonal anti-FLAG M2 antibody (1∶5,000 dilution in 5% nonfat milk in PBS) for 1 hour at 37°C and then washed 3× with PBS. Supersignal West Femto chemiluminescent substrate (Pierce-Thermo Fisher Scientific) was then applied to each well and signals were detected and quantified using a multi-well plate compatible luminometer (Glomax).

### Data Analysis

Signals from the target protein bands were quantified using ImageJ image processing and analysis software (National Institutes of Health, Bethesda, MD, http://rsbweb.nih.gov/ij/). Statistical analyses were performed using Microsoft Excel or GraphPad Prism 4 software (GraphPad Software, Inc.). Images were collected using exposure settings that did not saturate any of the pixels acquired by the camera. The signals resulting from detergent-soluble and insoluble preparations of a protein, respectively, were expressed as a fraction of the total signal and Student's t-test for independent means of unequal variance was used to determine if the amounts of signal from the target protein bands in each experimental group were significantly different. When testing the significance of means for more than 2 experimental groups, one-way analysis of variance (ANOVA) was used to first determine group statistical significance and only followed by Tukey’s post-hoc analysis if the initial comparison was found to be significant.

## Supporting Information

Figure S1 **TX100 solubility of Gβ1 is not affected by coexpression of D2R.** **A.** Representative image of Western blots depicting the segregation of transiently expressed Gβ1 into TX100-soluble (S) and insoluble (I) biochemical fractions (upper panels) or total cellular protein (lower panels) in HEK293 cells and the effect of transiently coexpressed D2R on this segregation. **B.** Quantification of the relative levels of Gβ1 segregating into TX100-soluble (white bars) and TX100-insoluble (black bars) biochemical fractions (mean ± SEM; n = 4).
(TIFF)

Figure S2 **Coexpression of Gβ5 does not affect the ability of D2R or MOR to translocate to the cell surface.** **A.** Quantification of the relative levels of cell surface D2R in HEK293 cells transiently transfected with a fixed amount of D2R cDNA and with cDNA for either Gβ1 or Gβ5. The cell surface D2R signal is expressed as a percent of the signal measured in cells transfected with the only the fixed amount of D2R cDNA. The levels of D2R specifically at the cell surface was evaluated by probing intact, non-permeabilized cells with anti-FLAG antibody targeting the D2R-fused extracellular N-terminal FLAG tag (mean ± SEM; n = 8–16, *p<0.01, Tukey’s post-hoc test, compared to cells expressing D2R alone). **B.** Quantification of the relative levels of cell surface MOR in HEK293 cells transiently transfected with a fixed amoun of MOR cDNA and with cDNA for Gβ5. The cell surface MOR is expressed as a percent of the signal measured in cells transfected with only the fixed amount of MOR cDNA. The levels of MOR specifically at the cell surface was evaluated by probing intact, non-permeabilized cells with anti-FLAG antibody targeting the MOR-fused extracellular N-terminal FLAG tag (mean ± SEM; n = 11–12).
(TIFF)

Figure S3 **Dopamine-mediated recruitment of Arr-BL to D2R-AP is blocked by treatment with staurosporine or partially blocked by overexpression of β-arrestin-2, as assessed by an in-cell “proximity biotin transfer assay.”** **A.** Representative images from a Western blot depicting total cellular biotinylated D2R-AP by coexpressed Arr-BL (top panels) and parent D2R-AP (bottom panels). The top left panel represents samples prepared from cells which were untransfected and treated with 10 µM biotin for 2 min (biotin +, first column) or samples that were transfected with both D2R-AP (+) and Arr-BL (+) and not treated with biotin (biotin −, second column); all following samples were transfected with D2R-AP (+) or Arr-BL (+) and treated with biotin (+). The third and fourth column in the leftmost panel represents basal levels of biotinylated D2R-AP (third) and the dopamine-dependent increase in biotinylated D2R-AP (fourth). The top center panel represents samples prepared from cells that were pre-treated for 10 min with 10 µM staurosporine (staurosporine +). The left column represents the D2R-AP biotinyaltion under staurosporine treatment and the right column represents the effect of dopamine in this condition. The top right panel represents samples prepared from cells which were also transfected with β-arrestin-2 in a 3∶1 ratio to Arr-BL (++arrestin), the left column represents the biotinylation of D2R-AP by Arr-BL, and the rightmost column represents the effect of dopamine on this condition. Biotinylated D2R-AP was detected by probing the blots with streptavidin. The bottom panels represent corresponding western blots from identical samples in the upper panel probed for the parent D2R-AP protein. **B.** Quantification of the relative levels of D2R-AP biotinylated by Arr-BL in response to dopamine treatment (10 µM for 30 min) in cells expressing only D2R-AP and Arr-BL, cells that were pre-treated for staurosporine, or cells transfected with 3∶1 β-arrestin-2: Arr-BL. Bars represent the dopamine-dependent percentage increase of biotinylated D2R-AP in each treatment condition (mean ± SEM; n = 4, *p<0.05, ANOVA followed by Tukey’s post-hoc test, compared to the increase in biotinylated D2R-AP in cells only treated with dopamine).
(TIFF)
