# Supplementary figures and images for: G Protein Beta 5 Is Targeted to D2-Dopamine Receptor-Containing Biochemical Compartments and Blocks Dopamine-Dependent Receptor Internalization
Source: PLoS One. 2014 Aug 27;9(8):e105791. doi: 10.1371/journal.pone.0105791 (PMC4146516; doi:10.1371/journal.pone.0105791)

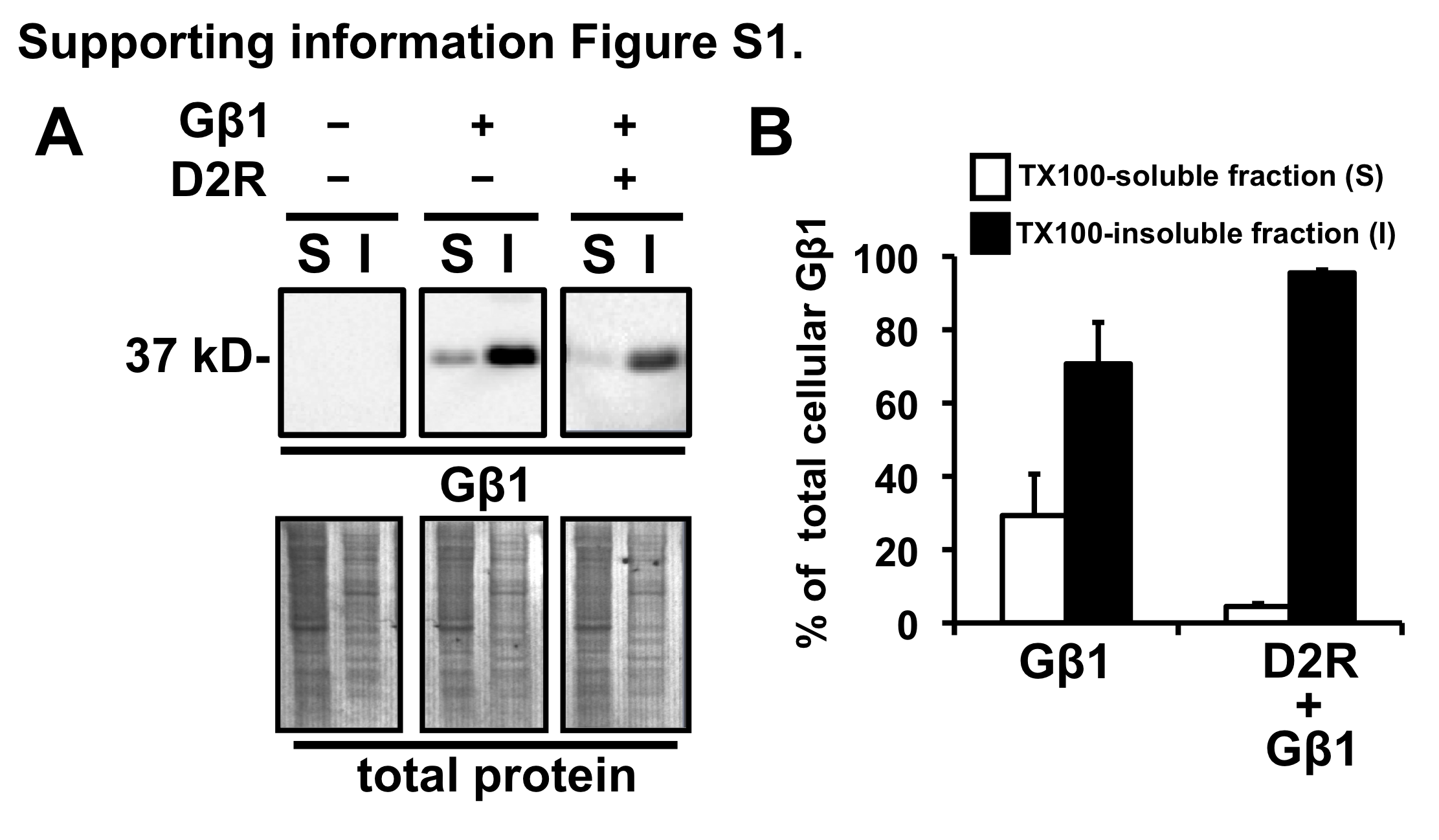

Supplement: Figure S1 — TX100 solubility of Gβ1 is not affected by coexpression of D2R. A. Representative image of Western blots depicting the segregation of transiently expressed Gβ1 into TX100-soluble (S) and insoluble (I) biochemical fractions (upper panels) or total cellular protein (lower panels) in HEK293 cells and the effect of transiently coexpressed D2R on this segregation. B. Quantification of the relative levels of Gβ1 segregating into TX100-soluble (white bars) and TX100-insoluble (black bars) biochemical fractions (mean ± SEM; n = 4). (TIFF) [file pone.0105791.s001.tiff]

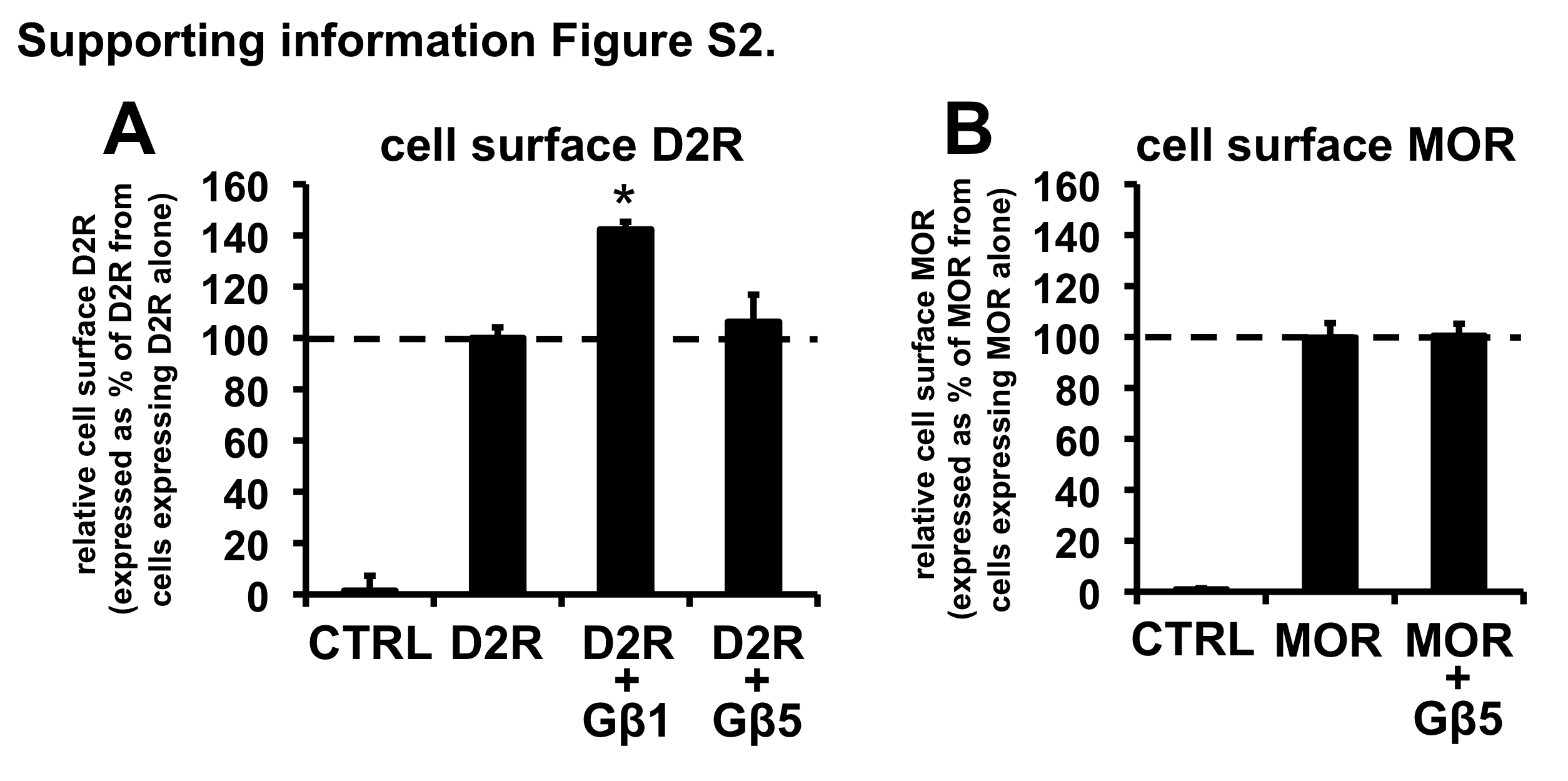

Supplement: Figure S2 — Coexpression of Gβ5 does not affect the ability of D2R or MOR to translocate to the cell surface. A. Quantification of the relative levels of cell surface D2R in HEK293 cells transiently transfected with a fixed amount of D2R cDNA and with cDNA for either Gβ1 or Gβ5. The cell surface D2R signal is expressed as a percent of the signal measured in cells transfected with the only the fixed amount of D2R cDNA. The levels of D2R specifically at the cell surface was evaluated by probing intact, non-permeabilized cells with anti-FLAG antibody targeting the D2R-fused extracellular N-terminal FLAG tag (mean ± SEM; n = 8–16, *p<0.01, Tukey’s post-hoc test, compared to cells expressing D2R alone). B. Quantification of the relative levels of cell surface MOR in HEK293 cells transiently transfected with a fixed amoun of MOR cDNA and with cDNA for Gβ5. The cell surface MOR is expressed as a percent of the signal measured in cells transfected with only the fixed amount of MOR cDNA. The levels of MOR specifically at the cell surface was evaluated by probing intact, non-permeabilized cells with anti-FLAG antibody targeting the MOR-fused extracellular N-terminal FLAG tag (mean ± SEM; n = 11–12). (TIFF) [file pone.0105791.s002.tiff]

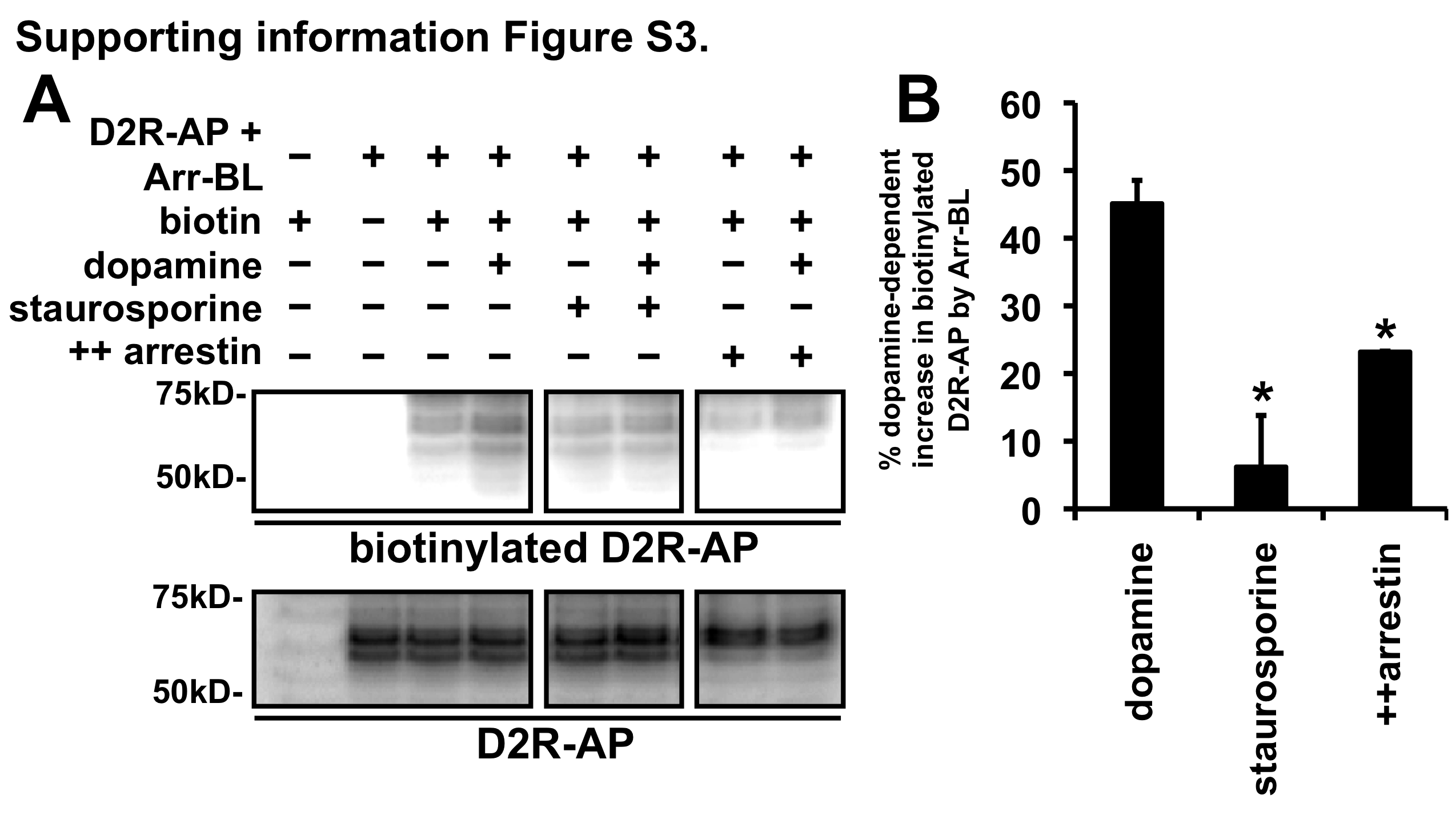

Supplement: Figure S3 — Dopamine-mediated recruitment of Arr-BL to D2R-AP is blocked by treatment with staurosporine or partially blocked by overexpression of β-arrestin-2, as assessed by an in-cell “proximity biotin transfer assay.” A. Representative images from a Western blot depicting total cellular biotinylated D2R-AP by coexpressed Arr-BL (top panels) and parent D2R-AP (bottom panels). The top left panel represents samples prepared from cells which were untransfected and treated with 10 µM biotin for 2 min (biotin +, first column) or samples that were transfected with both D2R-AP (+) and Arr-BL (+) and not treated with biotin (biotin −, second column); all following samples were transfected with D2R-AP (+) or Arr-BL (+) and treated with biotin (+). The third and fourth column in the leftmost panel represents basal levels of biotinylated D2R-AP (third) and the dopamine-dependent increase in biotinylated D2R-AP (fourth). The top center panel represents samples prepared from cells that were pre-treated for 10 min with 10 µM staurosporine (staurosporine +). The left column represents the D2R-AP biotinyaltion under staurosporine treatment and the right column represents the effect of dopamine in this condition. The top right panel represents samples prepared from cells which were also transfected with β-arrestin-2 in a 3∶1 ratio to Arr-BL (++arrestin), the left column represents the biotinylation of D2R-AP by Arr-BL, and the rightmost column represents the effect of dopamine on this condition. Biotinylated D2R-AP was detected by probing the blots with streptavidin. The bottom panels represent corresponding western blots from identical samples in the upper panel probed for the parent D2R-AP protein. B. Quantification of the relative levels of D2R-AP biotinylated by Arr-BL in response to dopamine treatment (10 µM for 30 min) in cells expressing only D2R-AP and Arr-BL, cells that were pre-treated for staurosporine, or cells transfected with 3∶1 β-arrestin-2: Arr-BL. Bars represent the dopamin [file pone.0105791.s003.tiff]
